# The Association of Body Size, Shape and Composition with Vertebral Size in Midlife – The Northern Finland Birth Cohort 1966 Study

**DOI:** 10.1038/s41598-019-40880-4

**Published:** 2019-03-08

**Authors:** Petteri Oura, Marjukka Nurkkala, Juha Auvinen, Jaakko Niinimäki, Jaro Karppinen, Juho-Antti Junno

**Affiliations:** 1 Medical Research Center Oulu, Oulu University Hospital and University of Oulu, P.O. Box 5000, FI-90014 Oulu, Finland; 2 Center for Life Course Health Research, Faculty of Medicine, University of Oulu, P.O. Box 5000, FI-90014 Oulu, Finland; 3 Research Unit of Medical Imaging, Physics and Technology, Faculty of Medicine, University of Oulu, P.O. Box 5000, FI-90014 Oulu, Finland; 4 Department of Sports and Exercise Medicine, Oulu Deaconess Institute, P.O. Box 365, FI-90101 Oulu, Finland; 5 Finnish Institute of Occupational Health, Aapistie 1, FI-90220 Oulu, Finland; 6 Cancer and Translational Medicine Research Unit, Faculty of Medicine, University of Oulu, P.O. Box 5000, FI-90014 Oulu, Finland; 7 Department of Archaeology, Faculty of Humanities, University of Oulu, P.O. Box 5000, FI-90014 Oulu, Finland

## Abstract

Small vertebral size increases the risk of osteoporotic vertebral fractures. Obese individuals have larger vertebral size and potentially lower fracture risk than lean individuals, but scarce data exist on the association between vertebral size and anthropometric measures beyond height, weight, and body mass index (BMI). Here, we evaluated several anthropometric measures (height, weight, BMI, waist circumference, hip circumference, waist-to-hip ratio [WHR], waist-to-height ratio [WHtR], fat mass [FM], lean body mass [LBM], percentage FM [%FM], percentage LBM [%LBM]) as predictors of vertebral cross-sectional area (CSA). We used a representative sample from the Northern Finland Birth Cohort 1966 (n = 1087), with anthropometric measurements from the ages of 31 and 46, bioimpedance analysis from the age of 46, and lumbar magnetic resonance imaging from the age of 46 years. In our data, height and LBM correlated most strongly with vertebral CSA among both sexes (0.469 ≤ *r* ≤ 0.514), while WHR, WHtR, %FM, and %LBM had the weakest correlations with vertebral CSA (|*r*| ≤ 0.114). We conclude that height and LBM have the highest, yet only moderate correlations with vertebral size. High absolute LBM, rather than FM or abdominal mass accumulation, correlates with large vertebral size and thus potentially also with lower osteoporotic vertebral fracture risk.

## Introduction

Vertebral fractures are the most common fragility fractures worldwide^1^. As vertebral size has a major influence on the biomechanical dispersion of loading forces across the vertebra, and thus also on its load-bearing capacity^2,3^, it is not surprising that small vertebral size seems to predispose individuals to vertebral fractures^4,5^. Relatively little, however, is known about the relationship between lifestyle factors and vertebral size, indicating that fracture risk assessment and fracture prevention may benefit from a more comprehensive knowledge of the potential lifestyle-related determinants of vertebral size. Interestingly, obese individuals have larger vertebral size^6^ and are potentially also at a lower osteoporotic vertebral fracture risk^7,8^ than lean individuals. In this study, we wanted to evaluate a wide range of ‘anthropometric measures’ (which we use here as a hypernym for anthropometric measurements and body composition parameters) that may be associated with vertebral size and thus influence vertebral fracture risk.

Anthropometric measurements quantify the shape and size of the body^9,10^ and body composition parameters estimate the components of body mass at the tissue level^11^. The skeleton develops in synchrony with the rest of the body^12^ which explains the strong correlation of height with skeletal robustness and thus also bone size^13^. Correspondingly, weight is associated with bone size at load-bearing skeletal sites^13^ and the dimensions of several bony elements from the axial and appendicular skeleton have been used to estimate height and weight to varying accuracies^14,15^. The lumbar vertebrae have substantial load-bearing responsibilities in the skeleton^2,16^, indicating that body size, shape, and composition may influence the vertebrae. Not surprisingly, previous studies have described positive associations between height, weight, body mass index (BMI, i.e. weight in kilograms divided by the square of height in meters) and vertebral size^5,6,17^.

Despite equal weight or BMI, different body shape and composition may influence the vertebrae differently depending on the distribution of mass in the body. When height and weight are assessed individually, any information on their reciprocal relationship, i.e. data on weight relative to height, is disregarded. Although BMI reflects this balance between height and weight, it omits the location and type of mass in the body^18,19^. Mass accumulation around the waist, hip, and abdomen are reflected by several anthropometric measurements, of which waist circumference (WC), hip circumference (HC), waist-to-hip ratio (WHR) and waist-to-height ratio (WHtR) are among the most widely used in clinical practice^18–21^. Of body composition parameters, fat mass (FM) and lean body mass (LBM, i.e. fat-free mass) represent the two main components of body mass^10,22^ and are therefore most typically assessed. While bone mass remains relatively constant in adulthood^23^, the amount and distribution of soft tissue may vary significantly over the life course. The vertebrae, as bony elements, may thus reflect LBM more strongly than FM or total body mass^22^. To date, however, data describing the association of vertebral size with anthropometric measures beyond height, weight, and BMI are scarce.

In this study, we aimed to evaluate several anthropometric measures as correlates of vertebral cross-sectional area (CSA) in a large general population sample of Northern Finns. First, we confirmed the previously established associations of adult height, weight, and BMI with midlife vertebral CSA, and then described the associations of other measures with vertebral CSA. Anthropometric measurements were objectively measured at the age of 31 (when peak bone mass had been newly reached^23^) and at the age of 46 (after which the incidence of osteoporotic vertebral fractures was known to increase^24,25^), body composition parameters were obtained by bioimpedance analysis at the age of 46, and vertebral CSA was derived from lumbar magnetic resonance imaging (MRI) scans at the age of 46.

## Methods

### Study population

The study was conducted using data from the Northern Finland Birth Cohort 1966 (NFBC1966). The cohort is described in more detail elsewhere^26^. In brief, the NFBC1966 is a prospective population-based birth cohort study which was initiated in 1966 when pregnant women and their children were recruited into the cohort (n = 12 231, corresponding to 96% of births in Northern Finland). NFBC1966 population members have been followed throughout their life course, with two main adult follow-ups at the ages of 31 and 46. At the age of 46, a representative subsample^27^ of the cohort underwent a lumbar MRI scan (n = 1540). The subsample was comprised of the cohort members who had participated in the previous data collections and lived within 100 km of the city of Oulu, Finland. In the present study, we excluded individuals with vertebral pathologies (n = 177) and missing anthropometric or body composition data (n = 276) from the MRI subpopulation, resulting in a sample size of 1087 individuals. This 46-year-old sample was considered relevant for the present study because the incidence of osteoporotic vertebral fractures begins to increase in late midlife^24,25^. Anthropometric variables from 31 years were also included because they represented the period of life when peak bone mass had been newly reached^23^; thereafter, bones have a limited ability to alter their geometry even if lifestyle factors such as body size or composition change drastically.

The study adhered to the principles of the Declaration of Helsinki with voluntary participation. All experiments were carried out in accordance with relevant guidelines and regulations. Informed consent was obtained from all participants. The data were handled on a group level and personal details were replaced by identification codes. The research was approved by the Ethics Committee of the Northern Ostrobothnia Hospital District.

### Vertebral size

**Figure 1:**
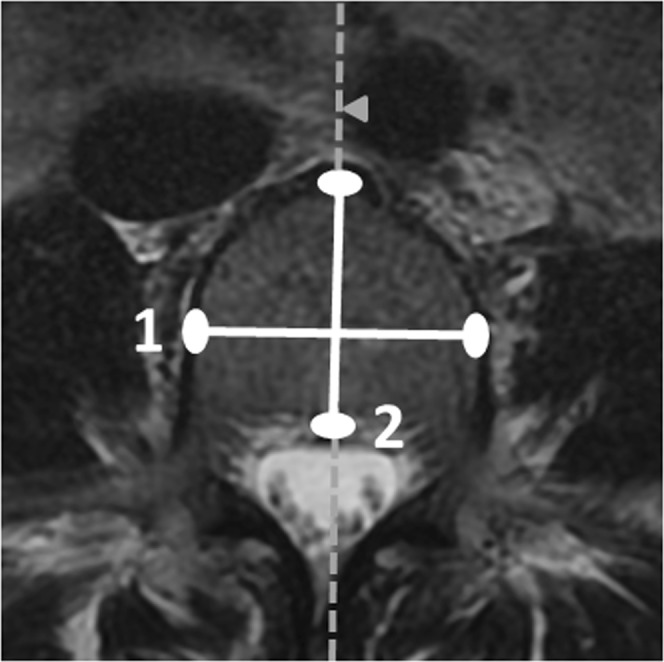
Annotated axial MRI scan of L4. Vertebral width = Measurement 1 and vertebral depth = Measurement 2. After all axial planes were examined, the maximum and minimum widths and the superior, inferior and midway depths were recorded.

The study participants’ vertebral size was measured from lumbar MRI scans. The scans were performed using a 1.5 T device (Signa HDxt, General Electric, Milwaukee, WI, USA) and a standard lumbar spine protocol (T2-weighted fast-recovery fast spin-echo images in sagittal and transverse planes), which is described in more detail elsewhere^26^. The scans were accessed using NeaView Radiology software version 2.31 (Neagen Oy, Oulu, Finland). After excluding vertebral pathologies, one researcher measured 1) the maximum and minimum widths of L4, and 2) the superior, midway, and inferior depths of L4, as illustrated in Fig. 1 (documentation accuracy of 0.1 mm). These vertebral measurements have also been presented in previous studies^17,26,28–32^. We chose L4 because it was located in the centre of the MRI scans and was known to accurately represent the other lumbar vertebrae^26,33^. In our previous studies, we have demonstrated the reliability and accuracy of our measurements (intraclass correlation coefficient = 0.963, mean directional measurement error 0.0% with a standard deviation of 4.9% around the mean)^26^ and shown that our MRI-derived measurements are equivalent to direct bone measurements^32^. The axial CSA of L4 was chosen to represent vertebral size as it is directly associated with vertebral load-bearing capacity and fracture risk^2–5^. CSA was calculated according to the formula^34^ CSA = π × mean width/2 × mean depth/2.

### Anthropometric measurements and body composition parameters

The following anthropometric measurements were systematically measured and documented during the 31- and 46-year follow-up examinations: height (accuracy of 0.1 cm), weight (i.e. total body mass, accuracy of 0.1 kg), WC (accuracy of 0.5 cm), and HC (accuracy of 0.5 cm). WC was measured from the middle of the lowest rib margin and the iliac crest, and HC was measured from the widest trochanters. Height and weight were measured using calibrated standard scales. BMI was calculated as weight (kg)/height squared (m^2^), WHR as WC (cm)/HC (cm), and WHtR as WC (cm)/height (cm).

At the 46-year clinical examination, body composition analysis was performed using the InBody 720 bioelectrical impedance analyser (Biospace Co., Seoul, Korea). FM (i.e. amount of fat, accuracy of 0.1 kg), LBM (i.e. fat-free mass, accuracy of 0.1 kg), and the corresponding percentages of total body mass (%FM, %LBM) were calculated from the data output. All measurements were taken after a 12-hour fasting period by a trained study nurse.

### Statistical analysis

We analysed the data using SPSS software version 24 (IBM, Armonk, NY, USA). The threshold for statistical significance was set at P = 0.05. We calculated descriptive statistics as means and standard deviations (SD), and analysed the sex differences at each time point using the independent samples t-test. The differences in each parameter between the time points were analysed using the paired samples t-test. We used Excel 2013 version 15.0 (Microsoft Corporation, Redmond, WA, USA) to create an exemplary scatter diagram illustrating the relationship between height and vertebral CSA. The associations between the anthropometric measures and vertebral CSA were evaluated using Spearman’s correlation (Spearman’s rho, *r*), as some of the anthropometric data were slightly right-skewed^35^. Separate analyses were performed for each anthropometric measurement taken at 31 and 46 years, and for each body composition parameter taken at 46 years. The correlation coefficients were interpreted according to Evans^36^: very weak to no correlation (0 ≤ |*r*| < 0.20), weak correlation (0.2 ≤ |*r*| < 0.4), moderate correlation (0.4 ≤ |*r*| < 0.6), strong correlation (0.6 ≤ |*r*| < 0.8), very strong correlation (0.8 ≤ |*r*| ≤ 1.0). All analyses were stratified by sex due to marked sex discrepancy in vertebral CSA.

## Results

A total of 490 men and 597 women had undergone clinical anthropometric measurements at the ages of 31 and 46, a bioimpedance analysis at the age of 46, and a lumbar MRI scan at the age of 46 years. Table 1 presents the characteristics of the sample. Compared to women, men had larger vertebral size and body size, as expressed by most anthropometric measures. Women, however, had higher FM and %FM (p < 0.001). Apart from height, all anthropometric measurements showed a clear increase between the ages of 31 and 46 among both sexes (p < 0.001).

**Table 1:** General characteristics of the sample. Values are presented as mean (standard deviation).

Characteristic	Men (N = 490; 45.1%)	Women (N = 597; 54.9%)	P
**Anthropometric measurements at age 31**			
Height (cm)	178.6 (6.2)	164.7 (5.7)	<0.001
Weight (kg)	79.7 (11.0)	65.0 (12.1)	<0.001
Body mass index (kg/m^2^)	25.0 (3.2)	24.0 (4.3)	<0.001
Waist circumference (cm)	88.2 (9.0)	77.9 (10.8)	<0.001
Hip circumference (cm)	96.9 (6.2)	96.6 (8.1)	0.471
Waist-to-hip ratio	0.91 (0.06)	0.80 (0.07)	<0.001
Waist-to-height ratio	0.49 (0.05)	0.47 (0.07)	<0.001
**Anthropometric measurements at age 46**			
Height (cm)	178.6 (6.2)	164.5 (5.7)	<0.001
Weight (kg)	86.0 (12.5)	71.6 (14.3)	<0.001
Body mass index (kg/m^2^)	26.9 (3.7)	26.5 (5.1)	0.075
Waist circumference (cm)	96.6 (10.4)	86.7 (12.7)	<0.001
Hip circumference (cm)	100.1 (6.5)	101.1 (10.7)	0.055
Waist-to-hip ratio	0.96 (0.06)	0.85 (0.06)	<0.001
Waist-to-height ratio	0.54 (0.06)	0.53 (0.08)	0.001
**Body composition parameters at age 46**			
Lean body mass (kg)	61.3 (6.7)	44.0 (5.3)	<0.001
Percent lean body mass (%)	72.4 (6.5)	63.0 (7.8)	<0.001
Fat mass (kg)	20.5 (8.5)	24.4 (10.8)	<0.001
Percent fat (%)	23.3 (6.8)	33.0 (8.3)	<0.001
**Lumbar magnetic resonance imaging at age 46**			
Exact age at imaging (years)	46.8 (0.4)	46.8 (0.4)	0.655
Cross-sectional area of L4 (cm^2^)	13.24 (1.74)	10.50 (1.31)	<0.001

L4 = fourth lumbar vertebra.

**Figure 2:**
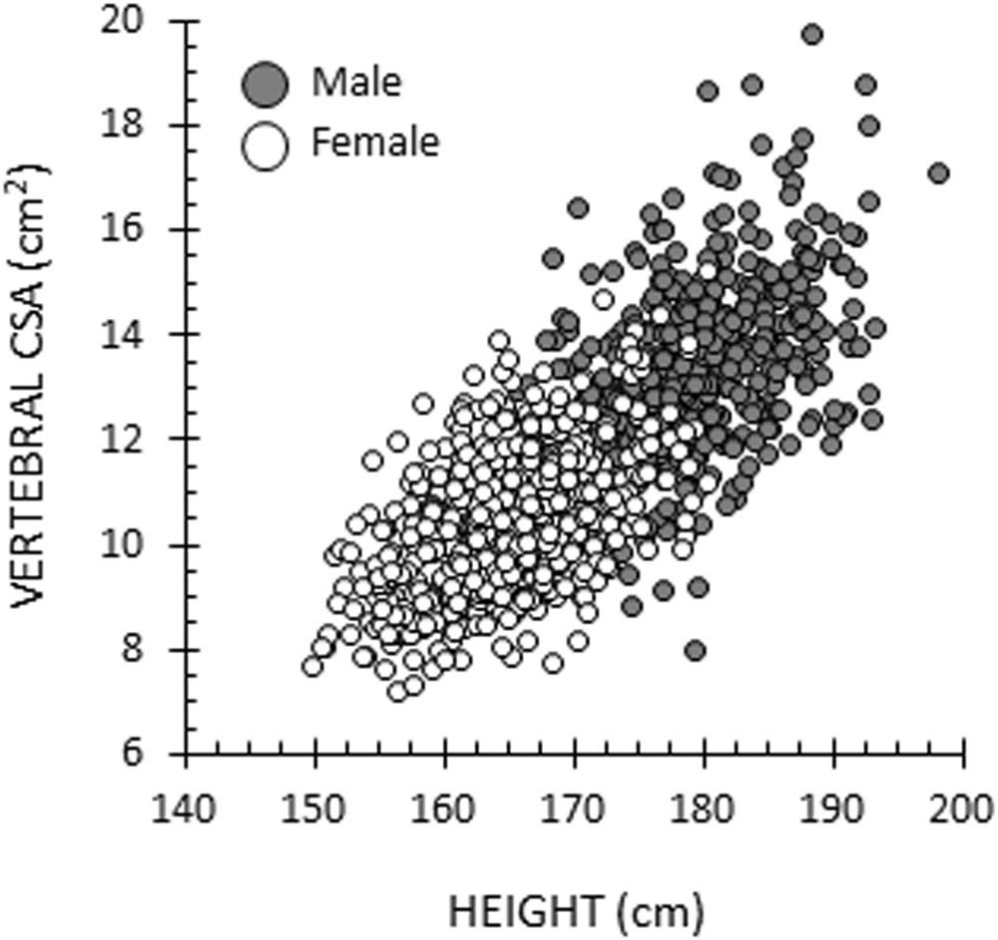
Scatter plot visualizing the correlation between height (measured at the age of 46) and vertebral cross-sectional area (CSA). Among men, *r* = 0.490 and among women, *r* = 0.482.

Tables 2 and 3 show the correlation coefficients between anthropometric measurements, body composition parameters, and vertebral CSA among the sample. Generally, the correlation coefficients ranged from −0.114 to 0.490 and −0.028 to 0.514 among men and women, respectively. Of the studied variables, height and LBM had the highest, yet only moderate, correlations with vertebral CSA (0.469 ≤ *r* ≤ 0.514). WHR, WHtR, %FM, and %LBM had the weakest correlations with vertebral CSA (|*r*| ≤ 0.114). Figure 2 is an exemplary scatter plot, demonstrating the relationship between height and vertebral CSA.

**Table 2:** Spearman’s correlation coefficients (*r*) for the relationship between anthropometric measurements (measured at the ages of 31 and 46) and vertebral cross-sectional area.

Parameter	Men		Women	
	*r*	P	*r*	P
**Height**				
At age 31	0.489	<0.001	0.480	<0.001
At age 46	0.490	<0.001	0.482	<0.001
**Weight**				
At age 31	0.362	<0.001	0.401	<0.001
At age 46	0.344	<0.001	0.320	<0.001
**Body mass index**				
At age 31	0.133	0.003	0.170	<0.001
At age 46	0.115	0.011	0.126	0.002
**Waist circumference**				
At age 31	0.163	<0.001	0.208	<0.001
At age 46	0.111	0.014	0.137	0.001
**Hip circumference**				
At age 31	0.290	<0.001	0.300	<0.001
At age 46	0.321	<0.001	0.235	<0.001
**Waist-to-hip ratio**				
At age 31	−0.035	0.435	0.040	0.333
At age 46	−0.114	0.012	−0.028	0.493
**Waist-to-height ratio**				
At age 31	−0.003	0.940	0.054	0.190
At age 46	−0.039	0.386	0.018	0.661

**Table 3:** Spearman’s correlation coefficients (*r*) for the relationship between body composition parameters (measured at the age of 46) and vertebral cross-sectional area.

Parameter	Men		Women	
	*r*	P	*r*	P
**Absolute values**				
Lean body mass	0.469	<0.001	0.514	<0.001
Fat mass	0.118	0.009	0.118	0.004
**Percentages**				
Percent lean body mass	−0.006	0.899	0.016	0.704
Percent fat	0.005	0.913	−0.016	0.688

## Discussion

Using a sample of 1087 individuals, this study aimed to investigate how several anthropometric measures (height, weight, BMI, WC, HC, WHR, WHtR, FM, LBM, %FM, %LBM) were associated with vertebral CSA in adulthood. According to the results, height and LBM were the strongest correlates of vertebral CSA among both sexes, whereas WHR, WHtR, %FM, and %LBM had the weakest correlations with vertebral CSA.

Obesity seems to reduce the risk of osteoporotic fractures^7^, although the association with vertebral fractures is less clear^8^. Yet, compared to lean individuals, those with a high BMI have larger vertebral CSA^6^, which increases the biomechanical dispersion of longitudinal loading forces across the vertebra and is therefore related to a higher vertebral load-bearing capacity^2,3^. Importantly, also the epidemiological literature suggests that large CSA is a protective factor against osteoporotic vertebral fractures^5^. Given these considerations, the results of this study not only provide knowledge on the association of body size, shape and composition with vertebral CSA, but may also benefit the assessment of osteoporotic vertebral fracture risk.

Previous studies have investigated the size of femur^37–39^ and other limb bones^22^ in relation to several anthropometric measures, and found varying positive correlations. Regarding the vertebrae, previous reports have described the correlation between vertebral dimensions and body height in adulthood (R = 0.2–0.6 for axial dimensions)^40,41^, weight in adulthood (R = 0.1–0.4)^41^ and in childhood (stated as ‘positive association’)^17^, and BMI across the lifespan (stated as ‘positive association’)^6^. As such, the present study is among the first to address the relationship between vertebral CSA and anthropometric measures beyond height, weight and BMI.

The anthropometric measures of our sample reflected a typical sex and age-related pattern^13,42^. Men had larger vertebral size, body size, lower body fat percentage, and a higher abdominal fat distribution than women. Age-related height loss was not yet present at 46 years of age, but a clear increase in body weight could be detected in both sexes over the follow-up. A similar pattern was visible in WHR, indicating that the age-related increase in weight was mainly a result of increased FM.

In our study, the anthropometric variables showed varying correlations with vertebral CSA (*r* = −0.114 to 0.514), and the results were similar among both sexes. As expected, adult height was among the strongest correlates of vertebral CSA (*r* = 0.480–0.490), most likely due to its role as a measure of overall skeletal size and thus bone size^13^. Despite the use of different vertebral measurements, the correlation was of similar magnitude to that of previous reports^40,41^. Of weight and its components (i.e. FM and LBM), LBM was found to predict vertebral CSA more strongly than total weight or FM (for LBM, *r* = 0.469–0.514; for total weight, *r* = 0.320–0.401; for FM, *r* = 0.118). Studies that have investigated femoral size have reported similar findings regarding LBM^37,39^. While total weight is influenced by changes in FM which may be subject to significant variation in a relatively narrow period of time, LBM remains more stable in this regard^22^. The skeleton has a limited ability to adapt to changes in body composition and lifestyle in terms of altering bone mass and geometry, which may explain the stronger association of LBM than FM or total weight with vertebral size.

Interestingly, %FM and %LBM had virtually no correlation with vertebral CSA (for %FM, *r* = −0.016–0.005; for %LBM, *r* = −0.006–0.016), suggesting that absolute body mass (i.e. higher LBM, FM and/or total weight) predicts vertebral CSA more strongly than percental body composition (i.e. the relationship between %FM and %LBM). Thus, fat percentage or LBM percentage seem to have little relevance by themselves; converting these into absolute mass values, i.e. accounting for total mass, seems to be necessary. A similar finding was also observed regarding WHR and WHtR, which had very weak or no correlation whatsoever with vertebral CSA (for WHR, *r* = −0.114–0.040; for WHtR, *r* = −0.039–0.054). As ratios of anthropometric measurements, WHR and WHtR contain little data on the absolute mass or size of an individual, and thus seem to be poor predictors of vertebral size. BMI, being essentially the ratio of weight and height, proved slightly better in predicting vertebral CSA (*r* = 0.115–0.170) but was still markedly less accurate than height or weight independently.

Waist and hip circumferences had weak to very weak correlations with vertebral CSA (for WC, *r* = 0.111–0.208; for HC, *r* = 0.235–0.321); curiously, the coefficients for HC were somewhat higher than those for WC. This is likely the result of the fact that HC is largely influenced by pelvic dimensions, which correlate with overall skeletal size and robustness^43^, whereas WC mostly reflects the amount of soft tissue in the abdominal area^42^.

The strengths of this study were its large representative sample, the anthropometric measurements which were objectively and systematically collected at two time points, and the body composition data which supplemented other measurements. Vertebral CSA data were systematically collected from lumbar MRI scans and had high reproducibility. The lumbar scans were taken at the age of 46 years which was considered a valuable time point for the assessment of vertebral size and fracture risk because the incidence of vertebral fractures begins to increase in late midlife^24,25^. Importantly, we excluded the lumbar scans that manifested vertebral pathologies. The study was thus able to focus on the healthy middle-aged vertebra in a well-characterized coeval sample. Anthropometric variables from 31 years were included because they represented the period of life when peak bone mass had been newly reached^23^.

This study also had limitations. Unlike the anthropometric measurements, body composition data were only available from the latter time point, i.e. from the age of 46 years. The lumbar MRI scans were also obtained at only one time point, which meant we could only assess association and not causality. Consisting of 46-year-old Northern Finns, our study population was geographically representative but somewhat problematic in terms of the wider generalizability of our results over other age groups or ethnical groups. Although the association between vertebral CSA and vertebral fracture risk has been investigated and stated in a number of previous articles^3–5^, we acknowledge that the aetiological basis of osteoporotic vertebral fractures is multifactorial. Our study is thus not conclusive, and our results need to be confirmed in future studies. Lastly, even though the present results demonstrate positive relationships between anthropometric parameters and vertebral size (i.e. large body size predicts vertebral robustness), the numerous negative health effects of excess weight^18^ need to be emphasized.

In this study, we assessed the association between several anthropometric measures and vertebral CSA in a large representative birth cohort sample. Of the studied variables, height and LBM had the highest, yet only moderate, positive correlations with vertebral size. Absolute LBM, rather than FM, %FM, or abdominal mass accumulation, correlated with vertebral size and thus potentially also with lower osteoporotic vertebral fracture risk. Further studies are needed to confirm our findings and investigate these anthropometric measures with respect to other bone outcomes.

## Data Availability

The datasets generated and analysed during the current study are not publicly available due to local privacy regulations but are available from the corresponding author on reasonable request.
